# Housing system and herd size interactions in Norwegian dairy herds; associations with performance and disease incidence

**DOI:** 10.1186/1751-0147-52-14

**Published:** 2010-02-16

**Authors:** Egil Simensen, Olav Østerås, Knut Egil Bøe, Camilla Kielland, Lars Erik Ruud, Geir Næss

**Affiliations:** 1 Norwegian School of Veterinary Science, Department of Production Animal Clinical Sciences, POB 8146 Dep, 0033 Oslo, Norway; 2 Norwegian University of Life Sciences, Department of Animal and Aquacultural Sciences, POB 5003, 1432 Ås, Norway; 3 North-Trøndelag University College, POB 2501, 7729 Steinkjer, Norway

## Abstract

**Background:**

According to the Norwegian animal welfare regulations, it has been forbidden to build new tie-stall barns since the end of 2004. Previous studies have shown that cow performance and health differ between housing systems. The interaction between housing system and herd size with respect to performance and disease incidence has not been evaluated.

**Methods:**

Cow performance and health in 620 herds housed in free-stall barns were compared with in 192 herds housed in tie-stall barns based on a mail survey and data from the Norwegian Dairy Herd Recording and Cattle Health Systems. The housing systems herds were comparable with respect to herd size (15-55 cows). Associations between performance/disease incidence and housing system, herd size and year of building the cow barn were tested in general linear models, and values for fixed herd size of 20 and 50 cows were calculated. On the individual cow level mixed models were run to test the effect of among others housing system and herd size on test-day milk yield, and to evaluate lactation curves in different parities. All cows were of the Norwegian Red Breed.

**Results:**

Average milk production per cow-year was 134 kg lower in free-stall herd than in tie-stall herds, but in the range 27-45 cows there was no significant difference in yields between the herd categories. In herds with less than 27 cows there were increasingly lower yields in free-stalls, particularly in first parity, whereas the yields were increasingly higher in free-stalls with more than 45 cows.

In free-stalls fertility was better, calving interval shorter, and the incidence rate of teat injuries, ketosis, indigestions, anoestrus and cystic ovaries was lower than in tie-stalls. All of these factors were more favourable in estimated 50-cow herds as compared to 20-cow herds. In the larger herd category, bulk milk somatic cell counts were higher, and the incidence rate of mastitis (all cases) and all diseases was lower.

**Conclusion:**

This study has shown that there is an interaction between housing system and herd size, and that performance and health is not universally better in small free-stalls than in tie-stalls.

## Background

Modern dairy production in large herds most commonly takes place in free-stall barns. Tie-stalls are, however, still an alternative to consider, particularly in smaller herds. In Norway tie-stalls constitute the most common housing system for dairy cows but, according to the Norwegian animal welfare regulations, it has been forbidden to build new tie-stall barns since the end of 2004. A panel appointed by European Food Safety Authority stated that there currently is limited amount of scientific data linking the period per day of being tied in tie-stalls to the level of disease and overall impact on welfare, so this should be studied. A minority of the panel recommended that dairy cattle should not be routinely kept in tie-stalls [1].

Previous studies have shown that performance and disease incidences vary between free-stalls and tie-stalls. A higher reproductive performance has been recorded in free-stalls [2-4] whereas milk yield has been reported to be lower [3-6]. Lower disease incidence rates have been recorded in free-stalls, i.e. of mastitis[2,6], teat injuries [4,7,8] and ketosis[2-4,9], whereas cows in free-stalls have shown poorer claw health [10-12]. Milk somatic cell counts have been found to be higher in free-stalls [5,13] or at the same level in the two housing categories [2,14]. When comparing housing systems, results may as well interact with differences in herd size. However, the interaction between housing system and herd size has not been studied and should be evaluated further.

The purpose of the present paper was to study differences in performance and disease incidences between free-stall and tie-stall housing for dairy cows in herds with approximately equal size, and to evaluate the interaction between housing system and herd size. The study is based on mail survey and data from the Norwegian Dairy Herd Recording System (NDHRS) and the Norwegian Cattle Health Recording System (NCHRS).

## Materials and methods

### Data

An overview of types of dairy cow housing systems (free-stalls or tie-stalls) was obtained by the NDHRS personnel from 11,600 dairy farms, which was 81% of all farms participating in the NDHRS. Fourteen per cent (1,600) of these farms were confirmed to have a free-stall system.

A questionnaire was sent to 2,400 farmers during March 2006. Of these 1,600 were confirmed as having free-stall barns and 800, with larger milk quota (more than 100,000 litres), where type of housing had not been confirmed. The purpose was to verify type of housing (by the farmer) and to obtain information on year of building, housing design and management in free-stall systems as basis for selection of farms for further studies. Questions were of the multiple-choice type, and alternative answers were pre-coded. Completed questionnaire was returned by 1,323 farmers (response rate of 55% after one reminder). Of these were 1036 farms with free-stalls and 287 with tie-stalls.

**Figure 1:**
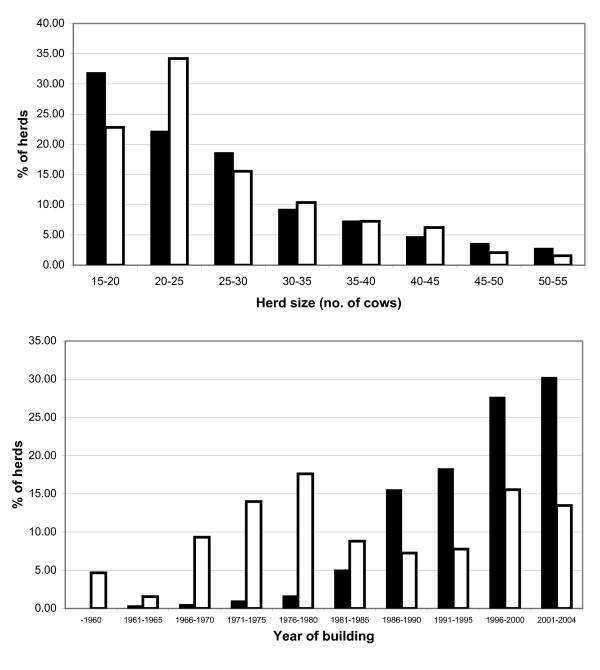
**Distribution of 620 free-stall and 192 tie-stall herds with respect to herd size (no. of cow-years) and building year of cow barn (building or last renovation)**. Black bars indicate free-stalls and white bars indicate tie-stalls.

The questionnaire data were merged with data from NDHRS [15], i.e. herd and individual cow level data for 2005 and 2006. Herds with barns built in 2005 were not included, since these herds were in a transition phase with respect to production. Furthermore, herds with other breeds than Norwegian Red Breed, an average of less than 3,500 kg milk per cow, less than 15 cows or less than 100,000 litres milk quota were also excluded. The maximum herd size was 53.9 cows in tie-stalls and 134.5 cows in free-stalls. In order to fit the material to a comparison between herd categories, free-stall herds with more than 55 cows (30 herds) were also excluded. The final data set included 620 free-stall herds (average herd size 26.5 cows, SD 9.4) and 192 tie-stall herds (average herd size 26.6 cows, SD 8.4). The average year of building or last renovation of the cow barns (representing the current building status) was 1984 (SD 14.3) for tie-stalls and 1996 (SD 7.2) for free-stalls. The distribution of herds with respect to herd size and year of building is shown in Fig. 1.

Comparison between free-stalls and tie-stalls at the herd level was based on NDHRS averages for the calendar year 2005. Studies at the individual cow level were performed using NDHRS data for 2005 and 2006. This material included cows that had terminated lactation during 2006. Cows with lactation periods of more than 483 days were excluded (2.5% of the lactations). Cows from breeds other than Norwegian Red were also excluded.

### Data extraction from NDHRS

The following data were extracted from NDHRS at herd level: Milk production per cow-year (estimated at mean production per 365 days), fertility index, herds mean calving interval, the herds annual geometric mean bulk tank milk somatic cell count (BMSCC).

The fertility index is defines as:

*Fertility status (FS-index)*: A measure of reproductive performance calculated according to the following formula:

where:

a = non return after 60 days (%) + % of 2 or 3 inseminations in same oestrus period,

b = number of services per inseminated cow or heifer,

c = average number of days between calving and last insemination,

d = number of cows culled because of infertility,

e = total number of inseminated cows or heifers.

Disease data at herd level were extracted from available calculated herd incidence rates estimated as cows having at least one event of disease or other events divided by number of cow years at risk, expressed as incidence rate per 100 cow-years. Separate incidences were available for removals, replacements, severe/moderate clinical mastitis, teat injuries, ketosis, milk fever, indigestion, retained placenta, abortion, anoestrus, metritis, cystic ovaries and all diseases.

At individual cow level, parity and calving interval for lactations that ended in removal or new calvings in 2006, altogether 26,276 lactations, was extracted. Furthermore test-days data regarding kg milk and information whether the cow was diseased or not on test-day were extracted. After removing all cows with other breeds than NRF, the data set was reduced to 25,898 lactations, of which 38% were cows in first parity, 26% in second, 18% in third and 19% in higher parities. Mean lactation length was 293 days (median 342 days, 25% with less than 223 days and 10% with less than 78 days and range 1-483 days). Of the included lactations, 14.5% were second lactation within the same cow. The test-day data which were extracted from these lactations included 191,046 observations from 21,235 cows. After removing test-say yields lower than 5 kg and higher than 60 kg, the final material included 185,272 observations.

### Statistical analyses

#### Herd level models

Statistical analyses were undertaken by means of SAS statistical package version 9.1 (SAS, Cary, NC, USA). Tests for herd level differences with respect to continuous variables, i.e. milk production per cow-year, fertility index, culling rate, calving interval and BMSCC were undertaken by means of general linear modelling (Proc GLM) after having tested for outliers and normal distribution. One model was made for each dependent variable separately:

X consisted of the independent variables stall type (1 = tie-stall, 2 = free-stall), herds size (continuous), the interaction between stall type and herd size and the year of building/reconstruction (continuous).

Differences between herd disease incidence rates were tested by means of general modelling (Proc GenMod in SAS) using the log link function and poisson or negative binomial distribution according to the best fit of the model assessed by deviance. Number of disease events in each herd were estimated from the given incidence rate and herd size (number of cow-years). Logarithm (Ln) of mean number of cows in the herd during one year was used as offset variable. If herd size was significantly associated with the outcome the model was adjusted for herd size. The model fit was evaluated by log likelihood or deviance. Independent variables were number of cases with the diseases mastitis, severe/moderate clinical mastitis, teat injuries, ketosis, milk fever, indigestion, retained placenta, abortion, anoestrus, metritis, cystic ovaries and all diseases. The model was E(Y) = nλ, where λ is a function of the predictors. Ln(λ) = β_0 _+ β_1 _X where variance = μ+ αμ^2 ^with a poisson distribution α = 1. X consisted of the independent variables stall type (1 = tie-stall, 2 = free-stall), herds size (continuous), the interaction between stall type and herd size or the year of building/reconstruction (continuous).

In all models at herd level non-significant variables were removed one by one, first the interaction between herd size and stall type and thereafter herd size or building year if non-significant. Significance was set at p ≤ 0.05, but the variable was included in the model if p < 0.10.

#### Test-day level model with milk yield as dependent variable

Finally a different test-day models was run using mixed models with herd as random effect and repeated measurements within lactation, nested within herd, applying an autoregressive type 1 regression (AR1) matrix. As kg milk was not log-transformed the model was estimating the lactation curve parameterized using the Wilmink equation [16]:

where DIM = days in milk, X = represent all other fixed affects type of stall (tie- = 1 or free-stall = 2), herd size (continuous), calving interval and interaction by herd size and type of stall, herd size and DIM, type of stall and DIM and calving interval and DIM applied, Zh = the herd random effect, Zl = the lactation repeated effect within herd using AR(1) correlation matrix, and e = random error effect. As this model has well known interaction between parity and DIM and lnDIM, the model was stratified on parity both to avoid random effect at individual and the interaction terms (parity and DIM) which would make the model too complicated to deduce.

## Results

Pairwise analyses by means of the analysis of variance showed that milk production per cow-year was 134 kg lower in free-stalls than in tie-stalls, 6943 vs. 7077 kg (p < 0.05). In free-stalls FS-index was higher than in tie-stalls (78.9 vs. 57.2, p < 0.001), calving interval was lower (369 vs. 384 days, p < 0.001), and the incidence rate of the following diseases were also lower: teat injuries (1.08 vs. 2.12, p < 0.001), ketosis (1.30 vs. 3.39, p < 0.001), indigestions (0.66 vs. 1.46, p < 0.001) and anoestrus (1.19 vs. 2.28, p < 0.002). No difference between housing systems was found for among others mastitis (all cases) all diseases and BMSCC.

**Figure 2:**
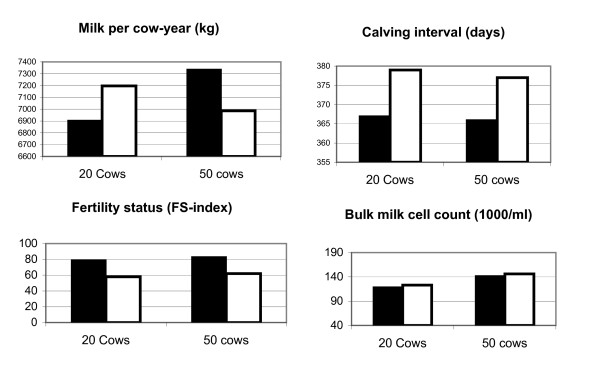
**Expected least squares means from model estimates (Table 1) at a herd size of 20 and 50 cows in 620 free-stall and 192 tie-stall herds**. Black bars indicate free-stalls and white bars indicate tie-stalls.

**Figure 3:**
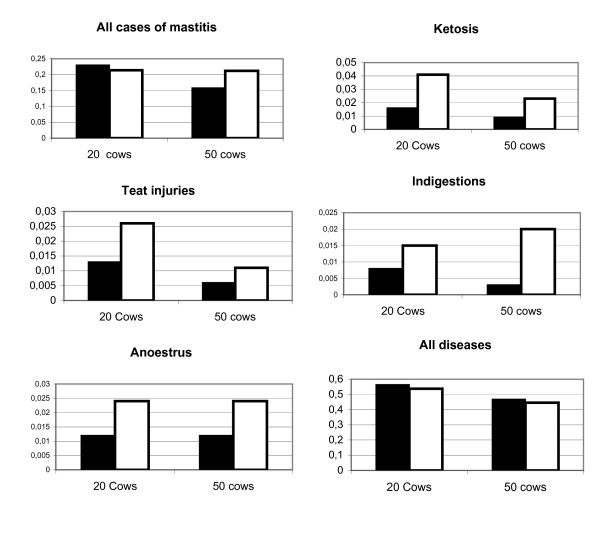
**Expected incidence rates from model estimates (Table 2) at a herd size of 20 and 50 cows in 620 free-stall and 192 tie-stall herd**. Black bars indicate free-stalls and white bars indicate tie-stalls.

The herd level models included herd size and year of building of the cow barn in addition to housing system, and the results regarding performance are shown in Table 1. Estimated means at a fixed herd size of 20 and 50 cows for factors being significantly associated with housing system and herd size in the models are shown in Fig. 2. Correspondingly, results from the models regarding disease incidences are shown in Table 2, and model estimates for 20- and 50-cow herds are presented in Fig. 3. The results are summarized in Table 3. Year of building of the cow barn had no significant associations with disease incidences, but it had a significantly negative association with calving interval. In free-stalls, the following factors were significantly different from tie-stalls: Lower milk yields, higher FS-index, lower calving interval, lower incidence rate of teat injuries, ketosis and anoestrus. Except from anoestrus, all of these factors were also associated with herd size, i.e. higher milk yields and higher FS-index, and lower incidences of teat injuries and ketosis in 50-cow herds as compared to 20-cow herds. The following significant associations were found with herd size, but not with type of housing: Higher BMSCC and lower incidences of mastitis (all cases), indigestions and all diseases in the 50-cow category.

**Table 1:** Model estimates (with standard error in brackets) from the general linear (GLM) model estimates in 620 free-stalls and 192 tie-stalls using Proc GLM in SAS.

	Intercept	Tie-stall	Herd size	Interaction tie-stall herd size	Year of building	Herd size in 2^nd ^power
Milk per cow-year (kg)	7472.1 (277.2)^xxx^	719.1 (218.9)^xx^	-45.48 (19.04)^x^	-21.38 (7.83)^xx^		0.855 (0.299)^xx^
FS status (FS-index)	95.201 (7.724)^xxx^	-21.278 (1.894)^xxx^	-1.159 (0.536)^x^			0.018 (0.009)^x^
Calving interval (days)	782.22 (137.43)^xxx^	11.541 (1.72)^xxx^	0.913 (0.429)^x^		-0.214 (0.069)^xx^	-0.014 (0.007)^x^
BMSCC (1000/ml)	80.364 (12.740)^xxx^	3.779 (3.144)^ns^	2.429 (0.882)^xx^			-0.024 (0.014)^0.06^

^x ^= p < 0.05, ^xx ^= p < 0.01, ^xxx ^= 0.001

(Effect of free-stall = 0 in the models).

**Table 2:** Model estimates (with standard error in brackets) from the poisson or negative binomial (NB) model estimates in 620 free-stalls and 192 tie-stalls cows using Proc Genmod in SAS.

Disease event	Intercept	Tie-stall	Herd size	Interaction tie-stall herd size	Dispersion (If NB fit)
Severe/moderate mastitis	-1.8233 (0.0918)^xxx^	0.0160 (0.0695)	-0.0060 (0.0031)^0.005^		0.4254 (0.0379)
All cases of mastitis	-1.2239 (0.0922)^xxx^	-0.3144 (0.2075)	-0.0124 (0.0032)^xxx^	0.0121 (0.0072)^0.09^	0.4188 (0.0325)
Teat injuries	-3.7139 (0.2233)^xxx^	0.6633 (0.1487)^xxx^	-0.0285 (0.0077)^xxx^		1.0733 (0.2602)
Ketosis	-3.7847 (0.2240)^xxx^	0.9649 (0.1520)^xxx^	-0.0189 (0.0076)^x^		1.8793 (0.2907)
Milk fever	-2.9452 (0.0391)^xxx^	0.0702 (0.0782)			0.2071 (0.0476)
Indigestions	-4.1680 (0.3249)^xxx^	-0.2089 (0.5558)	-0.0303 (0.0115)^xx^	0.0391 (0.0189)^x^	1.2651 (03637)
Retained placenta	-3.3586 (0.0539)^xxx^	-0.0812 (0.1126)			0.6680 (0.1071)
Abortions	-6.0659 (0.1622)^xxx^	-0.5325 (0.4113)			Poisson
Anoestrus	-4.4459 (0.1232)^xxx^	0.7085 (0.2334)^xx^			5.8928 (0.8265)
Metritis	-4.4742 (0.0859)^xxx^	-0.4554 (0.2016)^x^			1.1197 (0.3160)
Cystic ovaries	-4.8557 (0.1151)^xxx^	-0.4537 (0.2151)^x^			3.1334 (0.6429)
All diseases	-0.4513 (0.0812)^xxx^	-0.0461 (0.0620)	-0.0062 (0.0028)^x^		0.4869 (0.0282)

^x ^= p < 0.05, ^xx ^= p < 0.01, ^xxx ^= 0.001

(Effect of free-stall = 0 in the models).

**Table 3:** Overview of significant associations between performance/disease incidences and type of housing/herd size (20 and 50 cows) as shown in Table 1 and 2 and Fig. 2 and 3.

	Housing system	Herd size	Interactions
Milk per cow-year	^xx^	^x^	^xx^
Fertility status (FS-index)	^xxx^	^x^	
Calving interval	^xxx^	^x^	
BMSCC		^xx^	
Mastitis, all cases		^xxx^	
Teat injuries	^xxx^	^xxx^	
Ketosis	^xxx^	^x^	
Indigestions		^xx^	^x^
Anoestrus	^xx^		
Metritis	^x^		
Cystic ovaries	^x^		
All diseases		^x^	

^x ^= p < 0.05, ^xx ^= p < 0.01, ^xxx ^= 0.001

**Figure 4:**
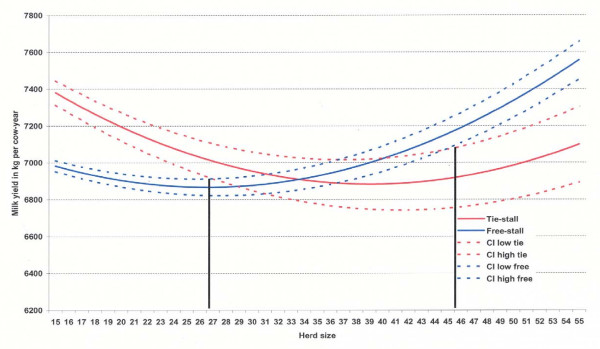
**Herd milk yields per cow-year and their 95% confidence intervals in 620 free-stall 192 tie-stalls related to herd size out from the general linear models as shown in Table 1**.

The interaction between housing system and herd size with respect to milk production per cow is shown in Fig. 4. The 95% confidence intervals for milk yield in free-stalls and tie-stalls overlapped in herds from 27 to 45 cows, i.e. there was no significant difference between housing systems in this range. Outside this range the differences were significant. In herds with less than 27 cows there were increasingly lower yields in free-stall herds, whereas the yield were increasingly higher in free-stall herds with more than 45 cows.

**Figure 5:**
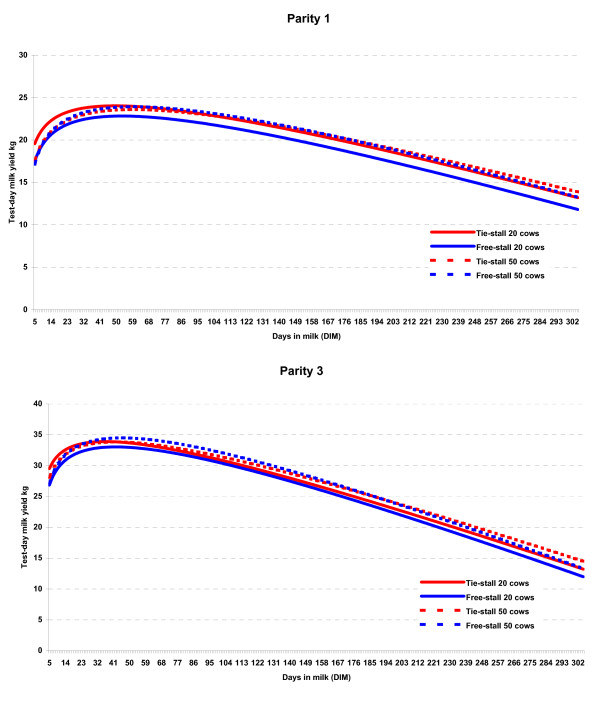
**Lactation curves for cows in parity 1 and 3 from model estimates (Table 4) in 662 free-stall and 192 tie-stall herds**.

The mixed models regarding test-day milk yields were run separately for each parity (1, 2, 3, >3). The lactation curves based on these models showed that the largest difference was found between parity 1 and 3. The results from the test-day models for these parities are shown in Table 4, and the lactation curves in Fig 5. The results were in agreement with the herd level model in that milk yields were significantly associated with housing system and herd size. In parity 1 the yields were lower in free-stalls in the 20-cow herds throughout the whole lactation, whereas they were at the same level in free-stalls and tie-stalls in 50-cow herds. In parity 3 the yields were also lower in the 20-cow herds, but the difference in relation to tie-stalls was less than for cows in parity 1. There was a difference in the shape of the lactation curve; yields being higher in the first 60-80 days of lactation in tie-stalls, and also in the last part of lactation. In the 50-cow herds the yields were higher in free-stalls in mid-lactation.

**Table 4:** Model estimates (with standard error in brackets) from the mixed models (ProcMixed in SAS) regarding test-day milk yield for cows in parity 1 (73,878 observations) and parity 3 (31,259 observations).

Parameter	Class	Estimate (β), parity 1	Estimate (β), parity 3
Intercept		10,9556 (0,4407)^xxx^	19,3046 (1,3906)^xxx^
Dim (days in milk)	Continuous	-0,08280 (0,001694)^xxx^	-0,1511 (0,004225)^xxx^
Lndim	Continuous	3,2422 (0,09681)^xxx^	5,0496 (0,2793)^xxx^
Housing (tie-stalls)	1	3,9006 (0,6385)^xxx^	5,4013 (0,9872)^xxx^
Housing (free-stalls)	2	0	0
Dim*housing	1	0,003912 (0,000921)^xxx^	0,009284 (0,001902)^xxx^
Dim*housing	2	0	0
Lndim*housing	1	-0,4517 (0,0763)^xxx^	-1,0271 (0,1472)^xxx^
Lndim*housing	2	0	
CI (calving interval)	Continuous	0,004768 (0,001409)^xxx^	0,008638 (0,001908)^xxx^
Dim*CI	Continuous	0,000043 (0.0000003)^xxx^	0,000101 (0,0000010)^xxx^
Lndim*CI	Continuous	0,0000007 (0,0000002)^x^	-0,00145 (0,000616)^x^
Dim* cows (herd no of cow-years)	Continuous	-0,00010 (0,000038)^xx^	-0,00018 (0,000079)^x^
Lndim*cows	Continuous	0,02167 (0,003022)^xxx^	0,02182 (0,006012)^xxx^
Cows	Continuous	-0,04516 (0,01325)^xxx^	-0,2022 (0,07613)^xx^
Cows*cows	Continuous	0,001119 (0,000142)^x^	0,002519 (0,001139)^x^
Cows*housing	1	-0,05597 (0,02091)^xx^	-0,05634 (0,03034)^ns^
Cows*housing	2	0	

^x ^= p < 0.05, ^xx ^= p < 0.01, ^xxx ^= 0.001

Random herd effect estimated to 19.0% in parity 1 and 19.8% in parity 3.

Random lactation effect estimated to 3.8% in parity 1 and 2.3% in parity 3.

## Discussion

The two herd categories differed with respect to year of building/renovation in that tie-stall buildings were in average 12 years older than the free-stall buildings. Four of the tie-stalls and 33 of the free-stalls were built in 2004 and were thus in a transition phase that year with respect to performance and health. We therefore used NDHRS data for 2005/2006 in the analyses. Other studies [5,17] have shown that cows changing from one system to another produced less milk the first few months after the transfer, but recovered after a full year. Automatic milking may be a confounding factor as it has been associated with a higher risk for high SCC [5], but in our material only 16 of the farms had automatic milking.

Usually herd estimates of cow performance are based on milk production per cow-year or disease incidences per cow-year as measures of intensity of milk production and disease occurrence at herd level. These measures are used as descriptive statistics, but they could be biased owing to different combinations of parities and different combinations of days in milk. This again could be due to different removal strategies in different herds. When analyzing the association between milk yield and housing system/herd size we therefore used two models. In addition to the herd level general linear model we also used individual cow test-day mixed models which also included calving interval in different parities. The results from these models confirmed the herd level result in that milk yields were significantly associated with housing system and herd size.

The finding that milk production per cow-year was lower in free-stalls is in agreement with results from previous studies in Norway [3,4] and Finland [5], whereas no difference was found between tie-stalls and loose housing in a large-scale study in Denmark [18]. Our result could indicate that free-stall barns have a less optimal function in smaller herds as other factors appear to intervene. Cattle are social animals and readily form dominance hierarchies, especially at areas of access to feed and water and the best resting area [19]. In general, changes in social strategies are related to variations in group size [20]. Sheep in larger groups spent less time queuing at the feed barrier [21], and in weaned pigs there were significantly more fights in smaller groups [22]. Among dairy calves increased group size reduced conflicts, and calves in the larger groups spent more time feeding [23]. Possibly similar interactions between group size and social behaviour also take place among adult cattle. The difference we found in the shape of the lactation curves, i.e. that yields were lowest among first parity cows primarily in 20-cow herds (Fig. 5) may indicate that these cows are lower in social rank and that this has larger consequences in smaller herds. The design of the cow barns may also be a factor having effect on cow behaviour, and suboptimal designs (area per cow, blind alleys, access to drinking water etc.) may have more unfavourable consequences in small free-stalls. Several of the associations recorded regarding performance and disease incidence were less favourable in the 20-cow herds as compared to the 50-cow herds, and this also indicates that small free-stalls are functioning less optimal.

The higher reproductive performance (higher fertility index, lower calving interval and lower treatments rates of anoestrus and cystic ovaries) which was found in free-stalls in this study is in agreement with previous results [2,3,9], and may possibly be related to easier oestrus detection when the cows are allowed to move freely. However, earlier oestrus detection and higher conception rates at first service have been observed in tie-stall barns as compared to free-stalls [24].

No significant difference between free-stalls and tie-stalls was found in average herd incidence rate of mastitis and BMSCC. This is in agreement with previous findings [3,4,7,25]. In another study, cows in tie-stalls had lower cell counts, but the proportion of cows having new high SCC for the first time and the incidence rate of mastitis did not differ between housing systems [5]. Lower mastitis incidence rate in free-stalls was found but no difference in somatic cell counts [2,6].

Teat injuries were found to be less frequent in free-stalls than in tie-stalls, and this is in accordance with previous results [4,7,8] Other studies have shown poorer claw health in free-stalls [10-12], but the present material did not provide sufficient information on claw health to make a comparison between housing systems possible.

The incidence rate of ketosis was 2.6 times higher in tie-stalls than in free-stalls, and this result is in agreement with previous findings [2-4,9]. The main risk factors for ketosis are related to nutrition and feeding. We found that the incidence of indigestions was higher in tie-stalls and that the shape of the lactation curve differed between herd categories, particularly in the first and last part of lactation for cows in parity 3 in the 20-cow herds. These results indicate that feeding strategies were different in the two housing categories.

## Conclusions

Several of the performance and health related factors were more favourable in free-stalls than in tie-stalls, and more so in estimated 50-cow herds as compared to 20-cow herds. Milk yields were increasingly lower in free-stall herds with less than 27 cows. Our study has shown that performance and health is not universally better in small free-stalls than in tie-stalls.

## Competing interests

The authors declare that they have no competing interests.

## Authors' contributions

ES, KEB and OØ had a central role in initiation of the research programme, CK, LER and GN carried out the mail survey, OØ performed the statistical analyses, and ES was the head writer of the manuscript. All authors have read and approved the final manuscript.
